# The Non-Receptor Protein Tyrosine Phosphatase PTPN6 Mediates a Positive Regulatory Approach From the Interferon Regulatory Factor to the JAK/STAT Pathway in *Litopenaeus vannamei*

**DOI:** 10.3389/fimmu.2022.913955

**Published:** 2022-06-29

**Authors:** Mengting Luo, Xiaopeng Xu, Xinxin Liu, Wenjie Shen, Linwei Yang, Zhiming Zhu, Shaoping Weng, Jianguo He, Hongliang Zuo

**Affiliations:** ^1^ State Key Laboratory of Biocontrol, School of Life Sciences, Sun Yat-sen University, Guangzhou, China; ^2^ Southern Marine Science and Engineering Guangdong Laboratory (Zhuhai), Zhuhai, China; ^3^ China-Association of Southeast Asian Nations (ASEAN) Belt and Road Joint Laboratory on Marine Aquaculture Technology, Sun Yat-sen University, Guangzhou, China

**Keywords:** non-receptor protein tyrosine phosphatase, antiviral immunity, IFN regulatory factor, JAK/STAT signaling pathway, *Litopenaeus vannamei*

## Abstract

SH2-domain-containing protein tyrosine phosphatases (PTPs), belonging to the class I PTP superfamily, are responsible for the dephosphorylation on the phosphorylated tyrosine residues in some proteins that are involved in multiple biological processes in eukaryotes. The Janus kinase/signal transducers and activators of transcription (JAK/STAT) pathway transduce signaling responding to interferons and initiate cellular antiviral responses. The activity of the JAK/STAT pathway is generally orchestrated by the de-/phosphorylation of the tyrosine and serine residues of JAKs and STATs, in which the dephosphorylation processes are mainly controlled by PTPs. In the present study, an SH2-domian-contianing PTP, temporally named as LvPTPN6, was identified in *Litopenaeus vannamei*. LvPTPN6 shares high similarity with PTPN6s from other organisms and was phylogenetically categorized into the clade of arthropods that differs from those of fishes and mammals. LvPTPN6 was constitutively expressed in all detected tissues, located mainly in the cytoplasm, and differentially induced in hemocyte and gill after the challenge of stimulants, indicating its complicated regulatory roles in shrimp immune responses. Intriguingly, the expression of LvPTPN6 was regulated by interferon regulatory factor (IRF), which could directly bind to the LvPTPN6 promoter. Surprisingly, unlike other PTPN6s, LvPTPN6 could promote the dimerization of STAT and facilitate its nuclear localization, which further elevated the expression of STAT-targeting immune effector genes and enhanced the antiviral immunity of shrimp. Therefore, this study suggests a PTPN6-mediated regulatory approach from IRF to the JAK/STAT signaling pathway in shrimp, which provides new insights into the regulatory roles of PTPs in the JAK/STAT signaling pathway and contributes to the further understanding of the mechanisms of antiviral immunity in invertebrates.

## Introduction

Protein tyrosine phosphatases (PTPs) are a sort of fundamental phosphatases responsible for the dephosphorylation of phosphorylated tyrosines in proteins that are involved in various biological processes including embryogenesis, organ development, tissue homeostasis, and the immune defenses in multicellular eukaryotes (1, 2). To date, more than 100 PTPs have been identified in the human genome and most of them possess non-redundant functions verified by the unique phenotypes of many reported gene deletions in mice (3, 4). According to the features of catalytic domains, PTPs can be classified into four subfamilies, among which classes I, II, and III are independently evolved PTPs that possess similar catalytic mechanisms and active targets, while class IV performs the catalytic function with a key aspartic acid and dependence on a cation (5–8). Class I PTPs are cysteine-based and strictly tyrosine-specific PTPs that possess the largest number of subfamily members, including the classical PTPs, vaccinia virus *H1*-like (VH1-like) enzymes, and “dual-specific” protein phosphatases (DSPs), constituting the most diverse group in terms of substrate specificity (9, 10). The classical PTPs can be further divided into receptor-like enzymes (RPTPs) and non-receptor PTPs (NRPTPs), which are distinguished by the containing of the transmembrane domain, while both of them are essential for the dephosphorylation of the tyrosine residues in their specific targets (5, 11).

The interferon (IFN) system plays a critical role in immunity, establishing a cellular antiviral state in cells of vertebrates and invertebrates (12, 13). The IFN regulatory factor (IRF) family is a kind of transcriptional factors, responsible for the transcriptional regulation of IFNs that can bind to IFN receptors and activate the Janus kinase (JAK)/signal transducers and activators of transcription (STAT) signaling pathway to promote the transcription of STAT-targeted genes (14, 15). The JAK/STAT pathway has been proved to mediate the transcription of numerous genes that participate in many cellular processes, such as cell proliferation, differentiation, apoptosis, and immunity, *via* a series of intracellular signaling cascades (16, 17). Briefly, after the binding of INFs, the transmembrane receptors are dimerized and trigger the auto- and trans-phosphorylation of JAKs to generate docking sites for the Src homology 2 (SH2) domains of latent STATs (18, 19). STATs are recruited and reversible phosphorylated by JAKs on a crucial tyrosine residue in their C-terminal region and then homo- or heterodimerized through the SH2-domain-phospho-tyrosine interactions. The activated STAT dimers are translocated into the nucleus for establishing the cellular transcriptional landscape (20, 21).

Since phosphorylation of tyrosine residues is a reversible process, activated STATs can be negatively regulated by PTP-mediated dephosphorylation. In mammals, seven PTPs had been proved to negatively regulate the JAK-STAT signaling pathway by dephosphorylating STATs, including PTP receptor-type D (PTPRD), PTP receptor-type T (PTPRT), PTP receptor-type K (PTPRK), Src homology region 2 (SH-2) domain-containing phosphatase 1 (SHP1, also named as PTPN6), SH-2 domain-containing phosphatase 2 (SHP2, also named as PTPN11), MEG2/PTP non-receptor type 9 (PTPN9), and T-cell PTP (TC-PTP)/PTP noc-receptor type 2 (PTPN2). However, some PTPs have been known to positively regulate the JAK/STAT signaling pathway through direct or indirect manners (22). For example, SHP-1 is positively involved in epidermal growth factor (EGF) and IFN-γ-induced STAT activation in non-hematopoietic Hela cells (23). SHP-2 could enhance the stability of JAK2 and facilitate prolactin/STAT5-mediated signaling by dephosphorylating tyrosine (Tyr-1007) of JAK2, a critical recruitment site for the ubiquitin ligase-associated inhibitory protein suppressor of cytokine signaling-1 (SOCS-1) (24).

Pacific white shrimp *Litopenaeus vannamei* is the major cultured shrimp species around the world, which has considerable nutritional and economic values (25). With the rapid development of the shrimp farming industry and the popularization of intensive farming systems, shrimp aquaculture has been threatened by various pathogens that cause huge economic losses (26, 27). Numerous studies had confirmed that *L. vannamei* possesses an IRF-Vago-JAK/STAT regulatory axis that is similar to the IRF-IFN-JAK/STAT axis of vertebrates, which establishes the important role of the JAK/STAT signaling pathway in shrimp anti-viral immunity (12, 28, 29). However, the detailed regulatory mechanism of the shrimp JAK/STAT signaling pathway remains unclear. In this study, the non-receptor protein tyrosine phosphatases 6 gene, temporarily named as LvPTPN6, was identified in *L. vannamei*. Results intriguingly revealed that LvPTPN6 mediated an intracellular IRF-JAK/STAT positive regulatory axis. These findings may provide new insights into the regulatory mechanism of the IRF-JAK/STAT signaling pathway in shrimp and give a better understanding of PTP evolution.

## Materials and Methods

### Shrimps

*L. vannamei* (∼10 g) were obtained from an aquaculture farm in Zhuhai, acclimated in a recirculating water tank system filled with air-pumped seawater (2.0% salinity), fed with 3% body weight artificial diet for two times each day, and detected by PCR to ensure they are free of WSSV, *Vibrio parahaemolyticus*, and *Staphylococcus aureus* with 5% random sampling.

### Cloning of LvPTPN6

Total RNA was extracted from mixed tissues including gill, hepatopancreas, intestine, and stomach sampled from healthy *L. vannamei* using an RNeasy Plus Mini Kit (QIAGEN, Hilden, Germany). The first-strand cDNA was synthesized using the PrimeScript™ II 1st-strand cDNA synthesis kit (Takara, Shiga, Japan) with DNase-treated total RNA as template and Oligo d(T)18 as a reverse transcript primer, and the open reading frame (ORF) of LvPTPN6 was amplified by primers of PTPN6-KpnIF and PTPN6-HA-PmeIR (**Table 1**). The 3′ and 5′ ends of LvPTPN6 mRNA were amplified by rapid amplification of cDNA ends (RACE) using a SMARTer RACE cDNA amplification kit (Clontech, Shiga, Japan) according to the manufacturer’s instructions as previously described (30).

**Table 1:** Primers and probes used in this study.

Name	Sequence (5′–3′)
**RACE**	
PTPN6-3′F1	ATGAGAACTCACAGTAGAAAAGATGATC
PTPN6-3′F2	TCGATTCTGTTGGTTTCTGTCTTCAG
PTPN6-5′R1	GATCATGGATTTGGGAAGATCTGTG
PTPN6-5′R2	AATTCACCTTTCTTGAGGCCTCATG
**Eukaryotic and prokaryotic expression vector**	
PTPN6-KpnIF	CGGGGTACCATGAGCTCGAGGAGATGGTTCCACCCG
PTPN6-HA-PmeIR	GGGTTTAAACTTAAGCGTAGTCTGGGACGTCGTATGGGTAGGTGGCACGGGGCGGCACAGATG
Dorsal-EcoRIF	TATGAATTCATCAAAATGTTTGTTGCCCAGCGTACTTCC
Dorsal-PmeIR	GGCGTTTAAACTTACATATCAGAAAATATCCAAAAC
Relish-EcoRIF	CAGGAATTCATCAAAATGGTGAGAGGTGACAGAGGTGG
Relish-XhoIR	ATACTCGAGCGCCTGGTCCAGTACAGCTACAC
STAT-XhoIF	ACTCTCGAGATCAAA ATGTCGTTGTGGAACAGAGC
STAT-V5-BstBIR	ACTTTCGAACTGAGGCTTCATGAAGTTGGTC
IRF-KpnIF	CATGGTACCATCAAA ATGCCGCCATCTTTCACCAATG
IRF-PmeIR	CATGTTTAAACTTACGGCAACGTCCTCTCGCCG
JAK-XbaIF	GTCTCTAGAATCAAA ATGCTGACAATCAGCTTCTACG
JAK-V5-BstBIR	GTCTTCGAACATGTACTCCTCTTGCAGTTCC
GFP-KpnIF	TGAGGTACCATCAAAATGGTGAGCAAGGGCGAGGAG
GFP-HA-PmeIR	CGAGTTTAAACTTAAGCGTAGTCTGGGACGTCGTATGGGTACTTGTACAGCTCGTCCAT
IRF-32a-EcoRIF	CCGGAATTCATGCCGCCATCTTTCACC
IRF-32a-NotIR	AAGGAAAAAAGCGGCCGCCTACGGCAACGTCCTCTCG
**EMSA**	
PTPN6 probe	/5Bio/ATAATGGTGATGATAGGTTGCTGGGGGAAAGGGGAAGGATCAAGGAGCAAGCGGAAGTGT
**Dual-luciferase reporter assays**	
PTPN6-pro-KpnIF	CGGGGTACCTGCATGTCAATGTATGAGTGTGTG
PTPN6-pro-HindIIIR	CCCAAGCTTCTCCAAACAAAATATCGACAATGA
PTPN6-p1-3′-KpnIF	CGGGGTACCGAGAAAATGTTGAGTGTGTGTG
PTPN6-p2-3′-KpnIF	CGGGGTACCAAAGATCTATTTAACTAGGTTAATG
PTPN6-p3-3′-KpnIF	CGGGGTACCTCACTTTCCCCGCCTTCGTG
PTPN6-p4-3’-KpnIF	CGGGGTACCTAAAATTATTATTATTTTTAAATTTAACC
ALF2-Pro-AscIF	AATGGCGCGCCGTGTACGTATGTATGTGTGTATGTG
ALF2-Pro-FseIR	ATTGGCCGGCCATGTGTTATGAATTGAAGTTCCTGAAG
ALF5-Pro-AscIF	AATGGCGCGCCCGCATATGTATGTATTCATGTATGTACAC
ALF5-Pro-FseIR	ATTGGCCGGCCGGAAGACGTGTTGTTGCTGTC
CTL4-Pro-SacIF	AATGGCGCGCCCTCTGAACAATGGCGGTTGAG
CTL4-Pro-FseIR	ATTGGCCGGCCGTTATCTGGTGAGAATGTGTCATG
Lys-IT2-SacIF	AATGGCGCGCCCAATTAAATTCTCCTTTGTATTGCACG
Lys-IT2-FseIR	ATTGGCCGGCCCCATGTGATGGGAGATCTACCTG
**dsRNA synthesis**	
PTPN6-dsT7F	GGATCCTAATACGACTCACTATAGGAGAAGTATGGTCGCATCACTGTCAG
PTPN6-dsR	CTGCTGCCTCACTGCTGTATTTG
PTPN6-dsF	AGTATGGTCGCATCACTGTCAG
PTPN6-dsT7R	GGATCCTAATACGACTCACTATAGGCTGCTGCCTCACTGCTGTATTTG
IRF-dsT7F	GGATCCTAATACGACTCACTATAGGGCCTTCAGTAGAACGCATAGAG
IRF-dsR	CCGTGCAGGTAGAGGTGGT
IRF-dsF	GCCTTCAGTAGAACGCATAGAG
IRF-dsT7R	GGATCCTAATACGACTCACTATAGGCCGTGCAGGTAGAGGTGGT
GFP-dsT7F	GGATCCTAATACGACTCACTATAGGATGGTGAGCAAGGGCGAGGAG
GFP-dsR	TTACTTGTACAGCTCGTCCATGCC
GFP-dsF	ATGGTGAGCAAGGGCGAGGAG
GFP-dsT7R	GGATCCTAATACGACTCACTATAGGTTACTTGTACAGCTCGTCCATGCC
**qRT-PCR**	
PTPN6-qRTF	GAGCATCAGGGTCCCACTATG
PTPN6-qRTR	GGCCTTCCTTGCGTGAGTAG
IRF-qRTF	GCATCTTCAGGATTCTGTGGAC
IRF-qRTR	AGAGCCCAGTAGCGAAAGAG
ie1-qRTF	GCCATGAAATGGATGGCTAGG
ie1-qRTR	ACCTTTGCACCAATTGCTAGTAG
ALF2-qRTF	TAGCGTGACACCGAAATTCAAG
ALF2-qRTR	CGAAGTCTTGCGTAGTTCTGC
ALF5-qRTF	TGGTGAAGGCTTCCTACAAGAG
ALF5-qRTR	CATCAGCAGTAGCAGTGTCAG
CTL4-qRTF	AACAAGCGGAGCAGTTCTG
CTL4-qRTR	CACAGCCAGTCACCTTCATAAG
Lys-IT2-qRTF	ACGCAGATAAGCCAATCATTGAG
Lys-IT2-qRTR	CAATCGTTCAGGAATTTAGCCATG
EF-1α-qRTF	TATGCTCCTTTTGGACGTTTTGC
EF-1α-qRTR	CCTTTTCTGCGGCCTTGGTAG

### Quantitative Real-Time PCR

For the transcriptional-level analysis of specific genes in *L. vannamei*, cDNAs were synthesized from total RNAs using PrimeScript RT reagent kit with gDNA eraser (Takara, Japan) according to the manufacturer’s instructions. Quantitative real-time PCRs (qRT-PCRs) were performed at a final volume of 10 μl containing 1 μl cDNA, 5 μl 2 × SYBR Premix Ex Taq II (Takara, Japan), and 0.5 μl each primer (10 μM) (**Table 1**) on a LightCycler 480 System (Roche, Germany). The optimized thermal cycling parameters were 95°C for 2 min to activate the polymerase, followed by 40 cycles of 95°C for 15 s, 60°C for 15 s, and 72°C for 15 s. Melting curves were generated by increasing the temperature from 72°C to 95°C (0.5°C/s) to denature the annealed DNA. The expression level of detected genes was determined using the 2^−ΔΔCt^ method after normalization to the internal control gene elongation factor 1 alpha (EF-1α, GenBank accession no:. GU136229) (31).

### Immune Challenge

For immune challenge, *V. parahaemolyticus* and *S. aureus* were cultured to the logarithmic phase and diluted to 10^5^ colony-forming units (CFUs) in 50 ml PBS after centrifugal collection, respectively. WSSV was prepared freshly from moribund shrimps infected with preserved WSSV stored at −80°C in our lab. Virus stock was quantified using absolute qRT-PCR and diluted to 10^6^ copes in 50 μl PBS as described previously (30).

For expression pattern analysis of LvPTPN6, healthy shrimps were divided into six experimental groups in independent recirculating water tank systems. After acclimation for 1 week, shrimps of six groups were injected with 10^6^ copies of WSSV, 10^5^ CFU of *V. parahaemolyticus* 10^5^ CFU of *S. aureus*, 5 μg of LPS, 5 μg of Poly(I:C), and PBS at the second abdominal segment, respectively. Hemocyte and gill were sampled from six randomly selected shrimps in each group at 0, 4, 12, 24, 48, 72, and 96 h post stimulant injection. After then, total RNAs were isolated and cDNAs were synthesized for qRT-PCR. For cumulative mortality analysis, shrimps (*n* = 40) were challenged with 10^6^ copies of WSSV at 48 h after the injection of LvPTPN6 or green fluorescent protein (GFP, as negative control) dsRNA. The cumulative mortality was recorded every 6 h after WSSV challenge. The WSSV copy number in muscle was detected at 3 and 5 days after WSSV challenge in parallel experiments. In each group, six living shrimps were randomly selected and the total DNA of muscle was extracted using DNeasy Blood and Tissue Kit (QIAGEN, USA), then the *ie1* (wsv069) DNA sequence (GenBank accession No. AY422228) was detected using relative qRT-PCR with shrimp EF-1α as internal control.

### Immunofluorescence

For subcellular localization analysis, the LvPTPN6 coding sequence was cloned into the pAc5.1 vector to express the HA-tagged LvPTPN6 protein. Drosophila Schneider 2 (S2) cells were plated on a siliconized coverslip with approximately 80% confluence, transfected with the pAc5.1-PTPN6-HA vector using FuGENE HD transfection reagent (Promega, Madison, WI, USA), and fixed with 4% paraformaldehyde for 5 min at 48 h post transfection. For nuclear translocation of *L. vannamei* STAT, shrimps were challenged with Poly(I:C) for 12 h as mentioned above, then hemolymph smear samples were made on siliconized slides and fixed with 4% paraformaldehyde for 10 min. For immunofluorescence assay, 4% paraformaldehyde fixed cells were infiltrated with 1% Triton X-100 for 20 min then successively incubated with rabbit Ab against HA (CST, Danvers, MA, USA) or shrimp STAT (GL Biochem, Shanghai, China) together with mouse Ab against β-actin (MBL, Tokyo, Japan) and Alexa Fluor 488-conjugated goat anti-rabbit Ab (CST, USA) together with Alexa Fluor 594-conjugated goat anti-mouse Ab (CST, USA). After staining with Hoechst 33342 (Invitrogen, Carlsbad, CA, USA) for the nuclei, sliders were observed using a TCS SP8 STED 3X Confocal Microscope (Leica, Munich, Germany). The immunofluorescence intensities of the cytoplasm- and nuclear-localized STAT were quantified and calculated using JACoP with an ImageJ plugin from four randomly selected microscopic vision fields in each group (32).

### RNA Interference *In Vivo*

To investigate the transcription and function of LvPTPN6, the selected transcription factors as well as LvPTPN6 were knocked down through RNA interference (RNAi). The templates of shrimp genes or GFP for dsRNA synthesis were cloned from the *L. vannamei* cDNA or pAc5.1-GFP plasmid and incorporated with T7 RNA polymerase promoter at the 5′ end using specific primes listed in **Table 1**, respectively. The dsRNAs were synthesized *in vitro* using the T7 RiboMAX™ Express RNAi system (Promega, USA), annealed from two independently transcribed single-strand RNAs, and then purified according to the manufacturer’s instruction. Acclimated healthy shrimps were divided into several groups (*n* = 40) and intramuscularly injected with 10 μg specific dsRNAs. Hemocytes and gills were sampled from nine shrimps in each group for the following mRNA and protein analysis at 48 h post-dsRNA injection as described above.

### Protein Preparation for Nuclear Localization Analysis of STAT

For nuclear localization analysis of STAT regulated by LvPTPN6 at the cellular level, S2 cells were co-transfected with pAc5.1-STAT and pAc5.1-PTPN6 or pAc5.1-STAT and pAc5.1-GFP (as control). Cells were collected at 48 h post-transfection followed by protein analysis as follows. For the analysis of nuclear localization of STAT, LvPTPN6 in *L. vannamei* were knocked down through RNAi as mentioned above. At 36 h post dsRNA injection, shrimps were challenged with Poly(I:C) for 12 h as mentioned above, then hemocytes were sampled from 30 randomly selected shrimps in each group followed by protein analysis. After preprocessing, the nuclear and cytoplasmic protein of S2 cells or hemocytes were isolated using NE-PER Nuclear and Cytoplasmic Extraction Reagents (Thermo, Waltham, MA, USA), respectively.

### Western Blot

For analysis of STAT localization, protein extractions were directly treated with SDS loading buffer to obtain protein samples, which were separated using 12% sodium dodecyl sulfate polyacrylamide gel electrophoresis (SDS-PAGE) and transferred to polyvinylidene difluoride (PVDF) membranes using the Trans-Blot Transfer system (Bio-Rad, Hercules, CA, USA) for the determination of the STAT level. For analysis of the STAT dimer level, S2 cells were lysed using Pierce IP Lysis buffer (Thermo, USA) at 48 h post-transfection to obtain the native proteins, which were separated with 10% native polyacrylamide gel electrophoresis and transferred to PVDF membranes in native transfer buffer. Western blot was performed using rabbit antibodies against shrimp STAT (GL Biochem, China), HistoneH3 (CST, USA), and β-actin (MBL, Japan), as previously described (33). HistoneH3 or β-actin was detected as an internal control of cytoplasmic or nuclear protein to rule out the possible contamination of nuclear or cytoplasmic protein, respectively. The protein levels were determined by analyzing the gray values of specific protein bands using Quantity One 4.6.2 software (Bio-Rad, USA) by the Gauss model and normalized to those of internal control.

### Co-Immunoprecipitation

The interactions between LvPTPN6 and *L. vannamei* JAK or STAT were analyzed using co-immunoprecipitation (Co-IP). The ORFs of STAT and *L. vannamei* JAK (JAK) were cloned into the pAc5.1/V5-His A plasmid (Invitrogen, USA) to generate V5-tagged STAT and JAK expression vectors, respectively. An HA-tagged GFP expression vector was used as control. HA-tagged LvPTPN6 or GFP and V5-tagged STAT or JAK were co-transfected into S2 cells. After 48 h, cells were harvested and lysed in IP Lysis Buffer (Thermo, USA) with a protease inhibitor cocktail (Sigma, St. Louis, MO, USA). Co-IPs were performed using anti-HA affinity agarose (Sigma, USA), and Western blot was performed with a rabbit anti-V5 primary antibody (Merck, Burlington, MA, USA) and a horseradish peroxidase (HRP)-conjugated goat anti-rabbit secondary antibody (Promega, USA).

### Dual-Luciferase Reporter Assay

For transcriptional regulation analysis of LvPTPN6, the promoter sequence of LvPTPN6 was retrieved from the genome data of *L. vannamei* (34) and completely or partly cloned into the firefly luciferase plasmid pGL3-Basic (Promega, USA) using primers listed in **Table 1** to generate pLG3-PTPN6, pGL3-PTPN6-p1, pGL3-PTPN6-p2, pGL3-PTPN6-p3, and pGL3-PTPN6-p4 recombinant plasmids. The ORF of transcription factors of *L. vannamei* including Dorsal, Relish, STAT, and IRF, as well as green fluorescent protein (GFP, set as negative control), was cloned into the pAc5.1-V5 plasmid (Invitrogen, USA) using specific primers listed in **Table 1** to obtain eukaryotic expression vectors, respectively. To verify the transcriptional activation of STAT on the anti-lipopolysaccharide factor (ALF), C-type lectin (CTL), and lysozymes (Lys), promoter sequences of ALF2, ALF5, CTL4, and Lys-IT2 were cloned into pGL3-Basic using primers listed in **Table 1** to generate pGL3-ALF2, pGL3-ALF5, pGL3-CTL4, and pGL3-Lys-IT2 recombinant plasmids. S2 cells were plated in a 96-well plate with 70% confluence and transfected with 50 ng pGL3 firefly luciferase plasmid, 30 ng pRL-TK Renilla luciferase plasmid (as an internal control) (Promega, USA), and 100 ng pAc5.1 expression vector mentioned, respectively. At 48 h post transfection, cells were lysed for examination of firefly and Renilla luciferase activities using a dual-luciferase reporter assay system (Promega, USA).

### Electrophoretic Mobility Shift Assay

To verify the regulatory role of LvIRF on LvPTPN6 transcription, the interaction between the LvIRF protein and LvPTPN6 promoter was detected through electrophoretic mobility shift assay (EMSA) *in vitro*.

LvIRF ORF was amplified using primers of IRF-32a-EcoRIF/NotIR, then cloned into the pET32a (Invitrogen, USA) prokaryotic expression vector; the original pET32a plasmid which expresses the thioredoxin (TRX) protein was set as negative control. The recombinant plasmids were transformed into *Escherichia coli* Rosetta (DE3) cells and induced with 1 mM isopropyl-beta-D-thiogalactoside (IPTG) in the logarithmic phase after enlarged cultivation. The His-tagged LvIRF or TRX protein was purified using Ni-NTA Agarose (QIAGEN, USA) and quantified using BCA Protein Assay Kit (Beyotime, Shanghai, China) according to the manufacturer’s instruction.

The 5′ biotin-labeled probe that contains the predicted IRF-binding motif sequence (GGAAAGGGGAAGGATCAA) was synthesized by Invitrogen (Shanghai, China) (**Table 1**). EMSA was performed using a LightShift Chemiluminescent EMSA Kit (Thermo, USA) as described previously (35). In general, the purified proteins (10 μg) were incubated with 20-fmol probes for the binding reactions between probes and proteins, separated by 5% native PAGE, transferred to positively charged nylon membranes, and cross-linked by UV light (256 nm). Then the biotin-labeled DNAs on the membrane were detected by chemiluminescence and captured by Amersham Imager 600 (GE, USA).

### Bioinformatics and Statistical Analysis

The IRF-binding motif in the LvPTPN6 promoter was predicted using JASPAR 2016 (http://jaspar2016.genereg.net/cgi-bin/jaspar_db.pl). The multiple-sequence alignment of PTPN6 homologs was performed using ClustalX 2.1, and the phylogenetic tree was constructed using MEGA 5.0. All data were presented mean ± SD. The significance of difference between groups of numerical data was calculated using Student’s *t*-test. The cumulative mortalities were analyzed using GraphPad Prism 5.01 to generate the Kaplan–Meier plot (log-rank χ^2^ test).

## Results

### Cloning and Bioinformatics Analysis of LvPTPN6

**Figure 1:**
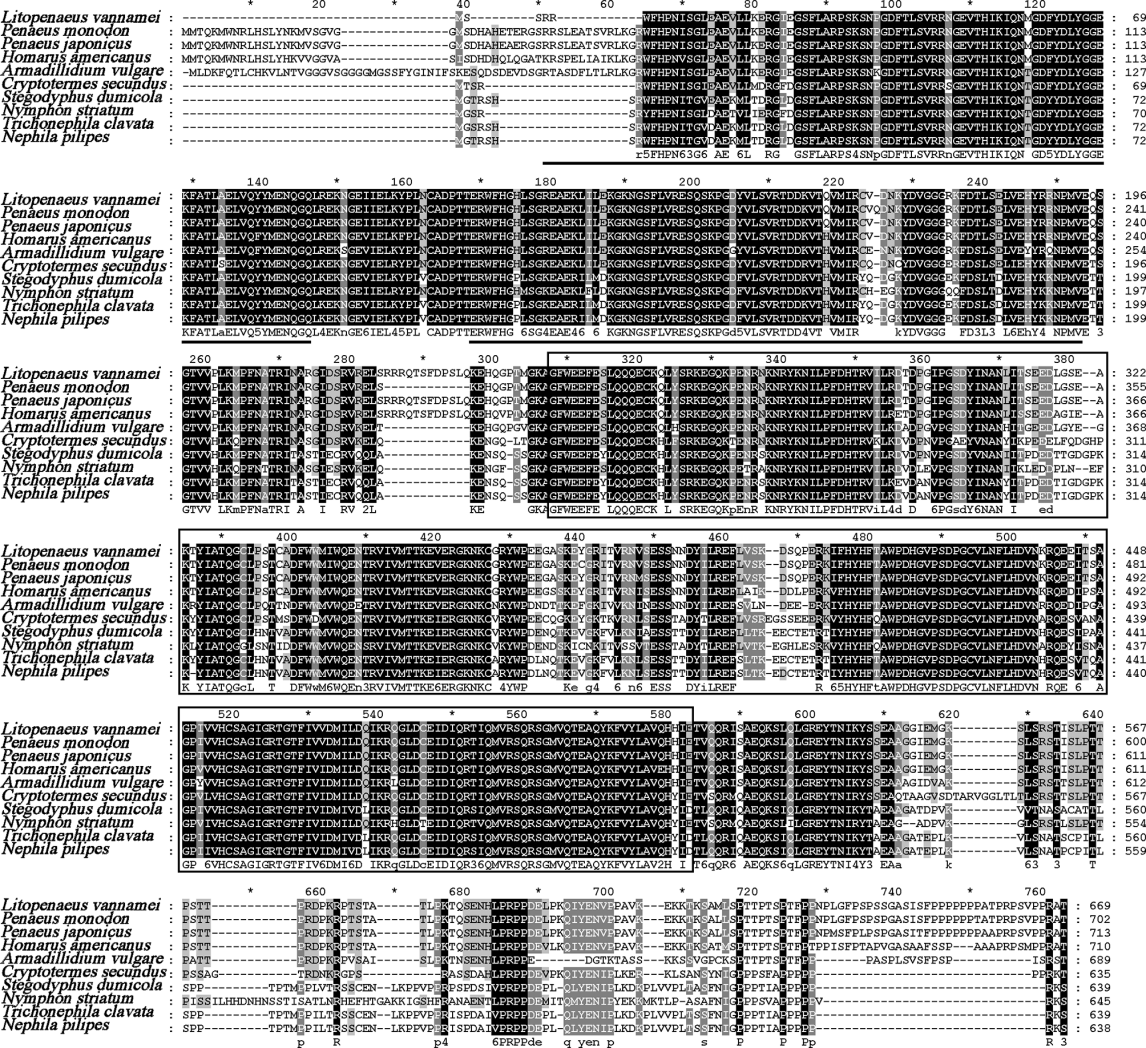
Multiple-sequence alignment of PTPN6 homologs. The two predicted SH2 domains were underlined, and the PTPc domain was framed. The amino acid sequence of PTPN6s was obtained from NCBI with GenBank accession numbers of *Penaeus monodon* (XP_037785856.1), *Penaeus japonicas* (XP_042879519.1), *Homarus americanus* (XP_042242946.1), *Armadillidium vulgare* (RXG72814.1), *Cryptotermes secundus* (XP_023713602.1), *Stegodyphus dumicola* (XP_035233634.1), *Nymphon striatum* (KAG1683061.1), *Trichonephila clavata* (GFR09275.1), and *Nephila pilipes* (GFS80663.1). The five tines star "*" means the middle position of the two Numbers beside it.

**Figure 2:**
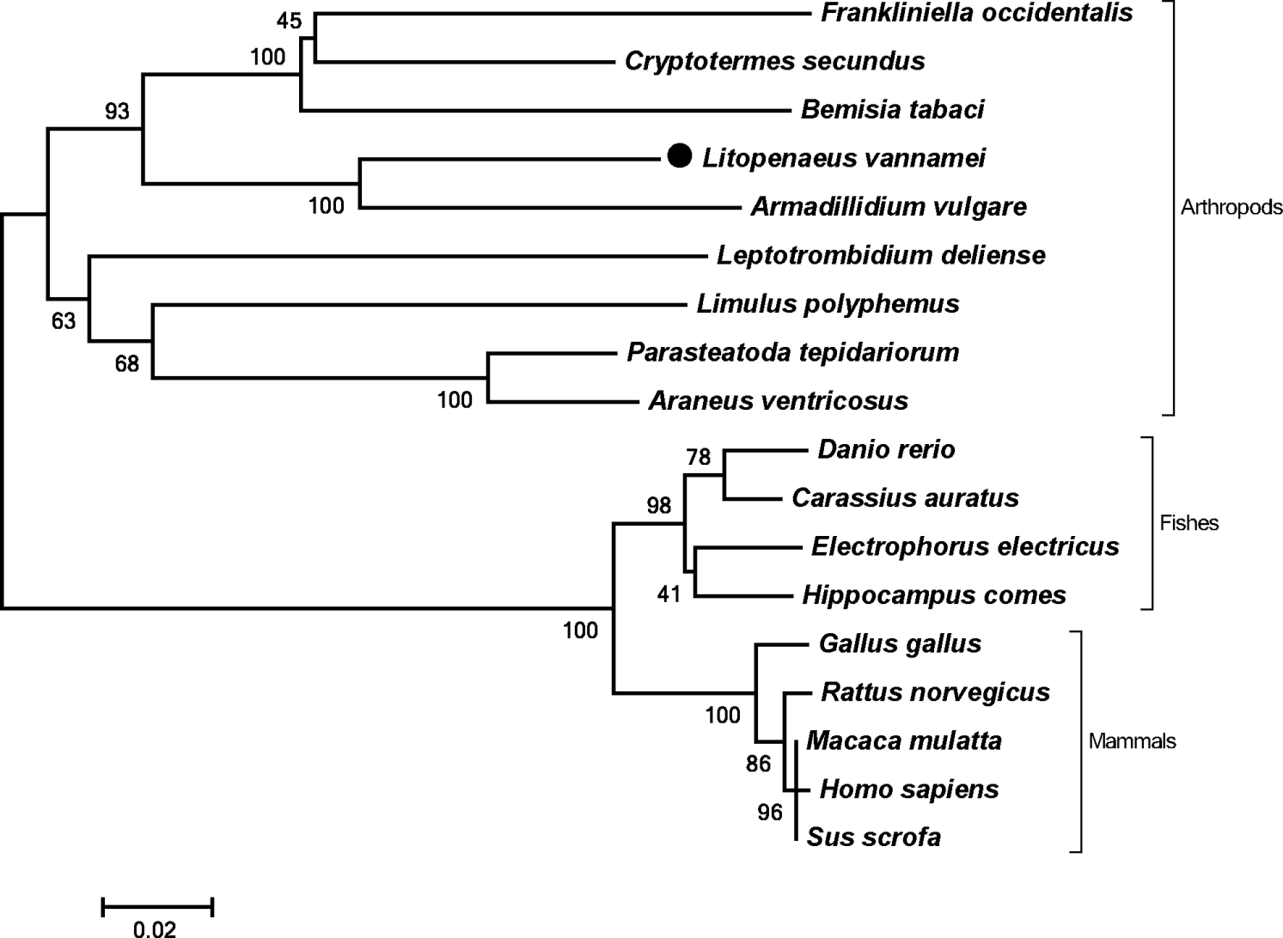
Phylogenetic tree of PTPN6s. Amino acid sequences of PTPN6s were obtained from NCBI with GenBank accession numbers of *Frankliniella occidentalis* (XP_026279043.1), *Cryptotermes secundus* (XP_023713602.1), *Bemisia tabaci* (XP_018912318.1), *Armadillidium vulgare* (RXG72814.1), *Leptotrombidium deliense* (RWS28580.1), *Limulus polyphemus* (XP_022245683.1), *Parasteatoda tepidariorum* (XP_015930014.1), *Araneus ventricosus* (GBM95823.1), *Danio rerio* (NP_956140.1), *Carassius auratus* (XP_026121403.1), *Electrophorus electricus* (XP_026888060.1), *Hippocampus comes* (XP_019712005.1), *Gallus gallus* (NP_990299.1), *Rattus norvegicus* (NP_001171064.1), *Macaca mulatta* (NP_001248038.1), *Homo sapiens* (NP_002822), and *Sus scrofa* (XP_013845818.1).

The full length of LvPTPN6 mRNA is 2,746 bp with a 5′ untranslated region (UTR) of 140 bp, an ORF of 2,010 bp encoding a protein of 669 amino acids, and a 3′ UTR of 596 bp (GenBank accession no.: OL652660). The LvPTPN6 protein has a calculated molecular weight of 75.62 kDa and a theoretical isoelectric point of 8.60 (**Supplemental figure 1A**). LvPTPN6 was predicted to contain two Src homology 2 (SH2) domains and a protein tyrosine phosphatase catalytic (PTPc) domain covering residues of 4–87, 110–193, and 248–519 (**Supplemental figure 1B**), which share the identical domain character as human PTPN6. Multiple-sequence alignment showed that LvPTPN6 shared high homology to the PTPN6s from Crustacea, Insect, and Arachnoidea, especially in the SH2 and PTPc domains, which shared higher identities of 98.2%, 96.15 and 92.2% with those of PTPN6s from *Penaeus japonicus*, *Penaeus monodon*, and *Homarus americanus*, and lower identities of 68.1%, 68.1%, 68.5%, and 69.9% from *Stegodyphus dumicola*, *Trichonephila clavata*, *Nephila pilipes*, and *Nymphon striatum*, respectively (**Figure 1**). In the constructed phylogenetic tree, the analyzed PTPN6s could be categorized into three clades of arthropods, fishes, and mammals (**Figure 2**), and LvPTPN6 was most closely clustered with the *Armadillidium vulgare* PTPN6 in the arthropod clade.

### Tissue Distribution and Subcellular Localization of LvPTPN6

**Figure 3:**
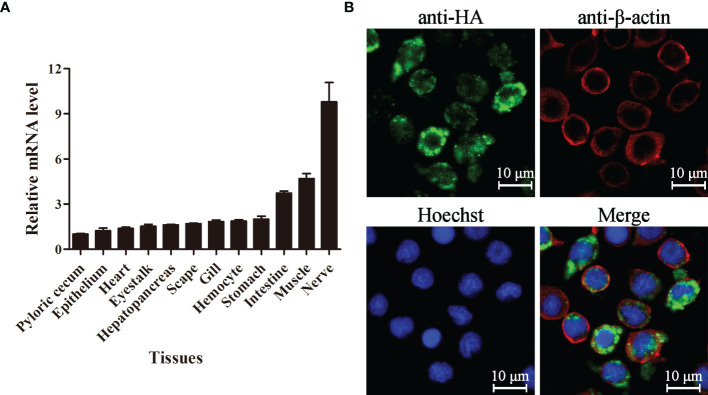
Tissue distribution and subcellular localization of LvPTPN6. **(A)** Expression of LvPTPN6 in *L. vannamei* tissues detected by qRT-PCR with EF-1α as internal control. The expression level of LvPTPN6 in pyloric cecum was set as the baseline (1.0). Each bar represents the mean ± SD (*n* = 4). **(B)** Subcellular localization of HA-tagged LvPTPN6 was detected by confocal laser scanning microscopy analysis in S2 cells. LvPTPN6 was stained with Alexa Fluor 488 (green), the cytomembranes were visualized by β-actin stain with Alexa Flour 594 (red), and the nuclei were stained with Hoechst 33342 (blue).

The expression level of LvPTPN6 in 12 tissues of *L. vannamei* was detected by qRT-PCR (**Figure 3A**). Results showed that LvPTPN6 could be detected in all selected tissues, and the lowest expression level was revealed in pyloric cecum. The expression level of LvPTPN6 was gradually increased in the epithelium, heart, eyestalk, hepatopancreas, scape, gill, hemocyte, and stomach, which was 1.22-, 1.39-, 1.53-, 1.62-, 1.71-, 1.85-, 1.87-, and 2.01-fold compared with it in pyloric cecum, respectively, and was roughly increased in the intestine, muscle, and the highest of nerve, which was 3.74-, 4.69-, and 9.80-fold compared with it in pyloric cecum. The subcellular localization of LvPTPN6 was detected using immunofluorescence assay in S2 cells which expressed the HA-tagged LvPTPN6 protein (**Figure 3B**). Results showed that LvPTPN6 was mainly located in the cytoplasm.

### Expression Profiles of LvPTPN6 After Immune Stimulation

**Figure 4:**
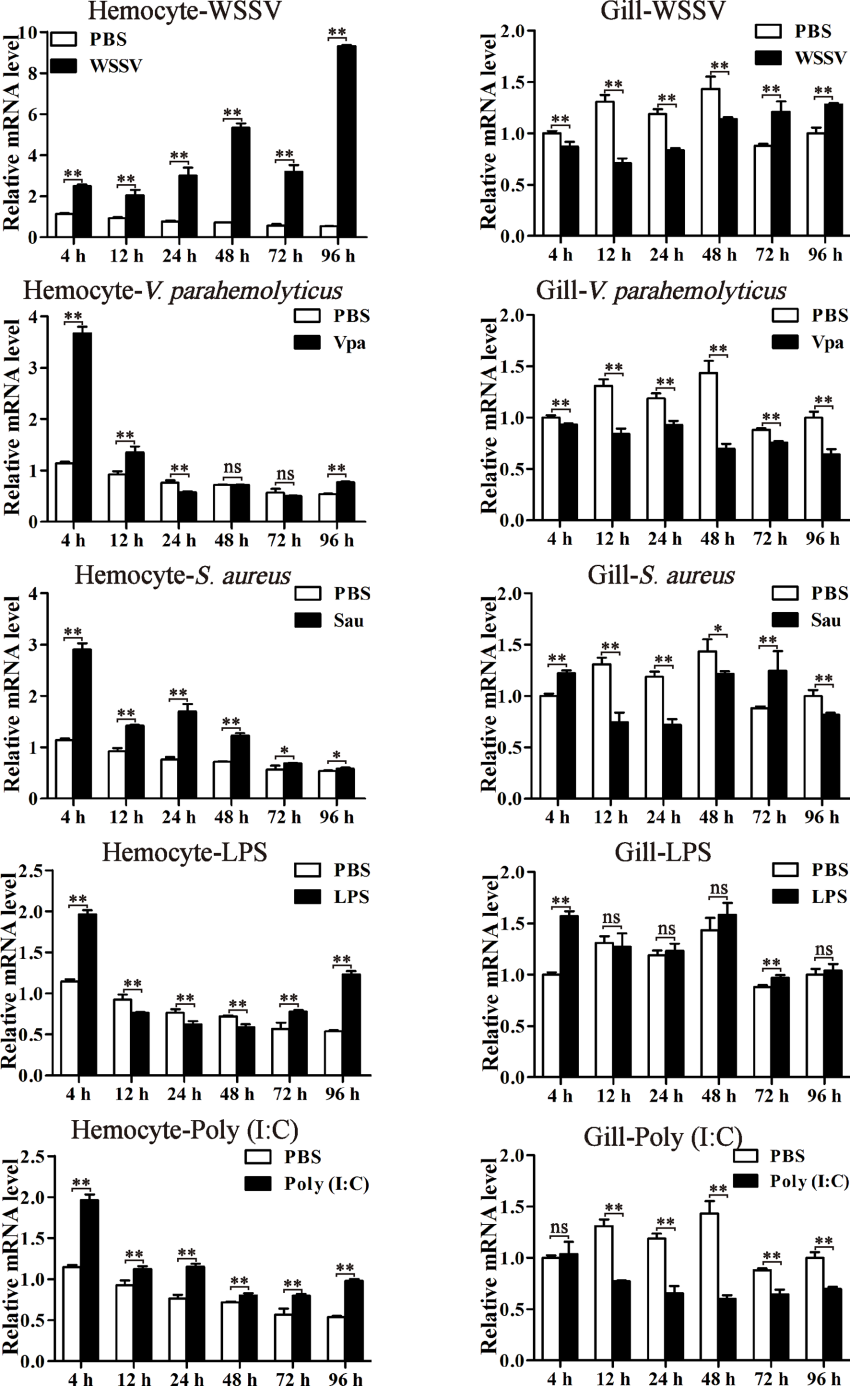
The expression profiles of LvPTPN6 after immune stimulation. The expression of LvPTPN6 in hemocyte and gill of WSSV-, *V. parahaemolyticus*- (Vpa), *S. aureus*- (Sau), LPS-, and Poly(I:C)-challenged shrimps were detected using qRT-PCR. In each panel, the value of PBS group at 4 h was set as the baseline (1.0). Each bar represents the mean ± SD (*n* = 4), **p < 0.01, *p < 0.05, and *ns* > 0.05 by two-tailed unpaired Student’s *t*-test.

The expression profiles of LvPTPN6 were investigated through qRT-PCR in hemocyte and gill from immune stimulated *L. vannamei* (**Figure 4**). In hemocyte, the expression levels of LvPTPN6 were generally upregulated after the stimulation of all stimulants. It was gradually increased from 1.18-fold at 4 h to 16.30-fold at 96 h after the stimulation of WSSV. However, it was roughly up-regulated of 3.2-, 2.05-, 1.71-, and 1.71-fold at 4 h, then gradually calmed down at the following time points, and up-regulated again of 1.43-, 1.90-, 2.29-, and 1.82-fold at 96 h after the stimulation of *V. parahaemolyticus*, *S. aureus*, LPS, and poly(I:C), respectively. Notably, the expression pattern of LvPTPN6 in gill was totally different from that in hemocyte, which was generally suppressed after the stimulation of WSSV, *V. parahaemolyticus*, *S. aureus*, and poly(I:C), while it did not significantly change, except being up-regulated of 1.57-fold at 4 h, after the stimulation of LPS.

### Regulation of LvPTPN6 Expression by IRF

**Figure 5:**
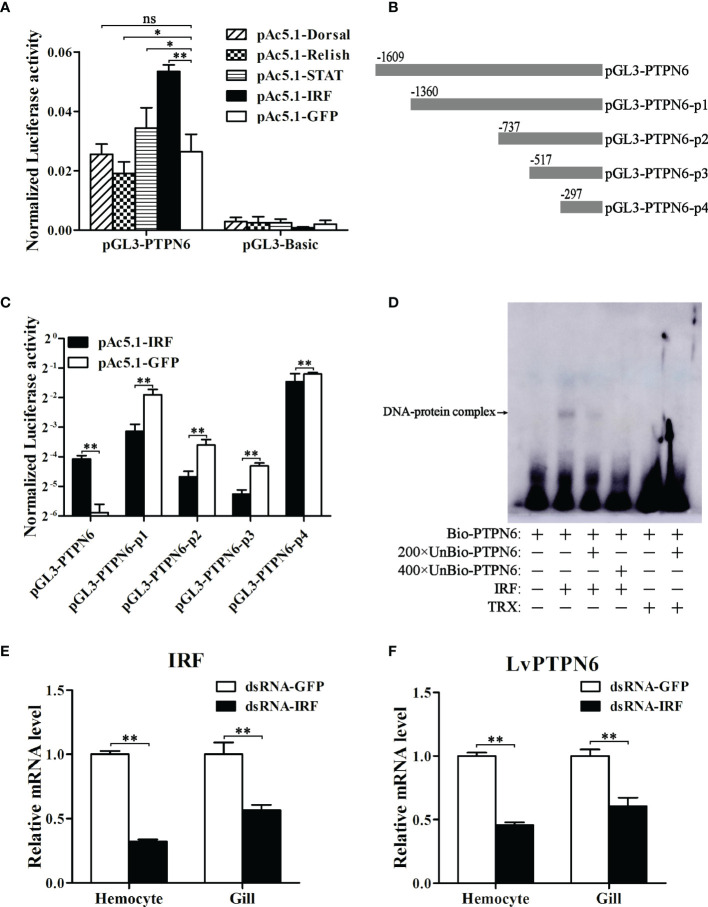
Regulation of LvPTPN6 expression by IRF. **(A)** Regulatory effects of Dorsal, Relish, STAT, IRF, and GFP (as control) on the LvPTPN6 promoter. Each bar represents the mean ± SD (*n* = 8), **p < 0.01, *p < 0.05, and *ns*: P > 0.05 by two-tailed unpaired Student’s *t*-test. **(B)** Scheme of the cleaved promoters of LvPTPN6. **(C)** Regulatory effects of IRF and GFP on the cleaved promoters of LvPTPN6. Each bar represents the mean ± SD (*n* = 8), **p < 0.01 by two-tailed unpaired Student’s *t*-test. **(D)** Interaction of IRF with the LvPTPN6 promoter analyzed by EMSA. The biotin-labeled (Bio-) or unlabeled (Unbio-) probes and purified prokaryotic protein of LvPTPN6 or TRX (as control) are used. **(E, F)** qRT-PCR analysis of IRF and LvPTPN6 mRNA levels in hemocyte and gill at 48 h post dsRNA injection. Values in the dsRNA-GFP control group were set as the baseline (1.0). Each bar represents the mean ± SD (*n* = 4), **p < 0.01 by two-tailed unpaired Student’s *t*-test.

A 1,609-bp promoter sequence (**Supplemental Figure 2A**) of LvPTPN6 was cloned into the pLG3-Basic vector and the transcriptional activity regulated by *L. vannamei* immune-related transcriptional factors, including Dorsal, Relish, STAT, and IRF, were analyzed through dual-luciferase assay. Results demonstrated that IRF could significantly up-regulate the transcription of the LvPTPN6 promoter of 2.03-fold compared with the GFP control (**Figure 5A**). To search the IRF-binding motif, the LvPTPN6 promoter was cleaved to -13,60, -737, -517, and -297 bp upstream of the transcriptional initiation site (**Figure 5B**) and cloned into the pGL3-Basic promoter. Results of dual-luciferase assay unveiled that the IRF-mediated transcriptional activation was vanished after the deletion of the -1,609 to -1,360 regions of the LvPTPN6 promoter (**Figure 5C**). In this region, a potential IRF-binding motif (GGAAAGGGGAAGGATCAA) was predicted through bioinformatics analysis (**Supplemental Figure 2B**), and its combination with IRF was verified *in vitro*. EMSA results showed that the IRF protein bound the biotin-labeled probe of the LvPTPN6 promoter to form a retarded shift band, which was gradually eliminated when 200 × and 400 × unlabeled probes were added to competitively bind IRF. In contrast, there was no shift band in the incubation between TRX protein and the biotin-labeled probe (**Figure 5D**). This suggested that LvPTPN6 could be directly regulated by IRF. To further verify the regulatory mechanism of LvPTPN6, IRF was silenced using the RNAi strategy *in vivo* (**Figure 5E**). Consistent with our expectation, silencing of IRF significantly inhibited the transcription of LvPTPN6 in both hemocyte and gill (**Figure 5F**), confirming that LvPTPN6 was transcriptionally regulated by IRF in *L. vannamei*.

### Interaction Between LvPTPN6 and JAK/STAT

**Figure 6:**
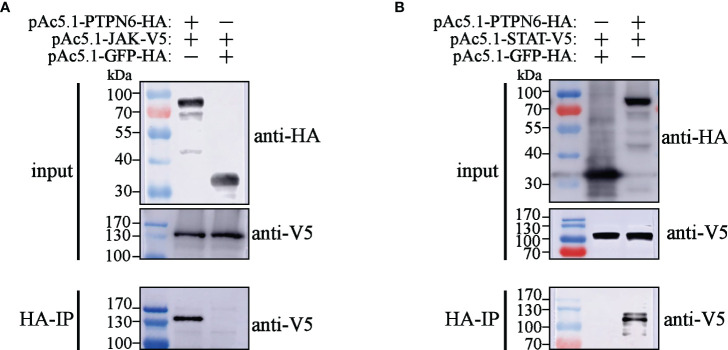
Co-IP analysis of the interaction between LvPTPN6 and JAK/STAT. Interaction between HA-tagged LvPTPN6 and V5-tagged JAK **(A)** or STAT **(B)**, HA-tagged GFP was set as internal control. Interacted proteins were precipitated by anti-HA affinity agarose.

To explore the mechanisms of LvPTPN6 promoting nuclear translocation of STAT, the interaction between LvPTPN6 and JAK/STAT was analyzed using Co-IP (**Figure 6**). Results showed that both V5-tagged JAK and STAT could co-precipitate with HA-tagged LvPTPN6 but not GFP protein, confirming the interaction between LvPTPN6 and JAK, and LvPTPN6 and STAT, respectively.

### Nuclear Localization of STAT Regulated by LvPTPN6

**Figure 7:**
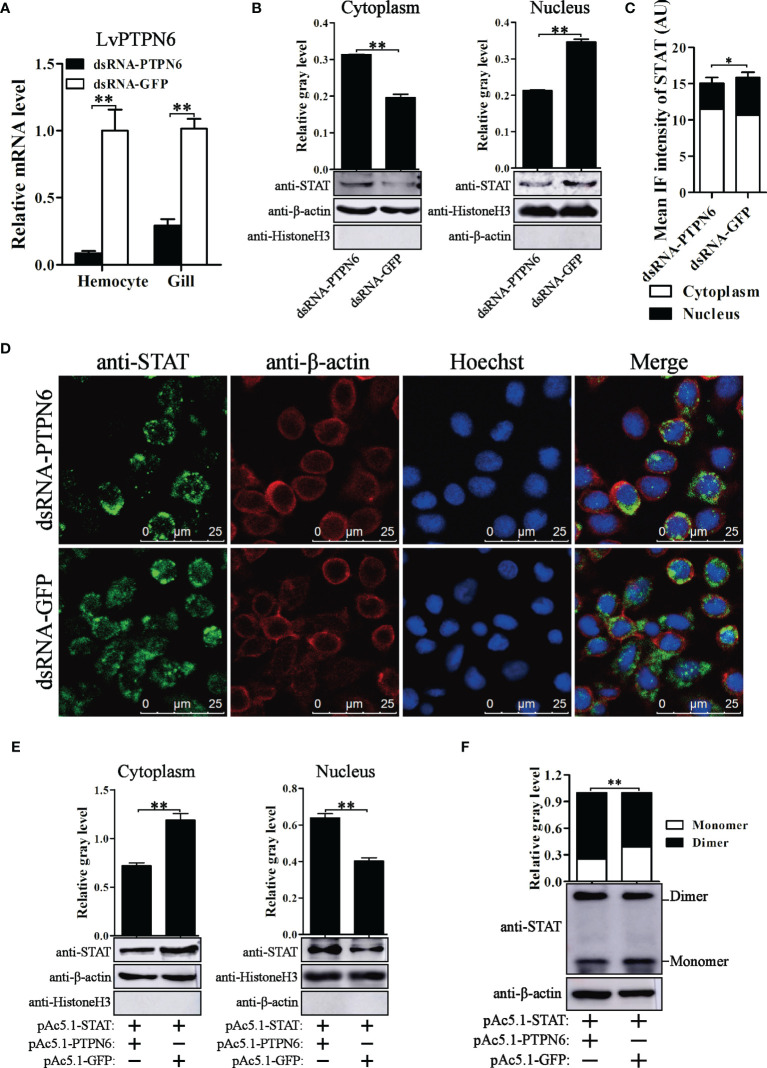
Nuclear localization of STAT regulated by LvPTPN6. **(A)** qRT-PCR analysis of the LvPTPN6 mRNA level in hemocyte and gill at 48 h post dsRNA injection. Values in the dsRNA-GFP control group were set as the baseline (1.0). Each bar represents the mean ± SD (*n* = 4), **p < 0.01 by two-tailed unpaired Student’s *t*-test. **(B)** Western blot analysis of the protein level of STAT located in the cytoplasm and nucleus of hemocytes after dsRNA injection. **(C)** Immunofluorescence intensities (arbitrary units, AU) of cytoplasm- and nuclear-localized STAT in dsNRA-GFP- and dsRNA-PTPN6-treated hemocytes. Immunofluorescence intensities were calculated using JACoP with an ImageJ plugin from four randomly selected microscopic vision fields (**Supplemental Figure 3**). *p < 0.05 by two-tailed unpaired Student’s *t*-test. **(D)** Immunofluorescent analysis of STAT in hemocytes after dsRNA injection. STAT was stained with Alexa Fluor 488 (green), the cytomembranes were visualized by β-actin stain with Alexa Flour 594 (red), and the nuclei were stained with Hoechst 33342 (blue). **(E)** Western blot analysis of the protein level of shrimp STAT located in the cytoplasm and nucleus of S2 cells overexpressed with LvPTPN6 or GFP (negative control). **(B, E)** In cytoplasm, the gray values of STAT bands were normalized to those of the cytoplasmic internal control of β-actin, and Histone H3 was detected to verify no contamination of nuclear protein. In nucleus, the gray values of STAT bans were normalized to those of the nuclear internal control of Histone H3, and β-actin was detected to verify no contamination of cytoplasmic protein. **(F)** Western blot analysis of the dimer and monomer levels of STAT through native PAGE after overexpressing LvPTPN6 in S2 cells. The gray values of STAT bans were normalized to those of the internal control of β-actin. **(B–F)** Each bar is mean ± SD of three independent quantification of the electrophoretic bands, **p < 0.01 by two-tailed unpaired Student’s *t*-test.

To reveal the regulatory role of LvPTPN6 on the JAK/STAT signaling pathway, nuclear localization of STAT was investigated after the down- or up-regulation of LvPTPN6 in *in vivo* or S2 cells, respectively. Western blot analysis showed that, after knockdown of LvPTPN6 and stimulation of Poly(I:C) *in vivo* (**Figure 7A**), cytoplasm-localized STAT was increased by 59.9%, and nuclear-localized STAT was decreased by 38.4% compared with the negative control in hemocyte, respectively (**Figure 7B**). This result was confirmed by immunofluorescence assay, which demonstrated that silencing of LvPTPN6 suppressed the nuclear translocation of STAT by 29.3% compared with the GFP control in hemocytes (**Figures 7C, D**). The facilitated role of LvPTPN6 on STAT nuclear localization was further verified through overexpression LvPTPN6 in S2 cells. Western blot analysis showed that overexpressed LvPTPN6 decreased the cytoplasm-localized shrimp STAT by 38.2% and increased the nuclear-localized shrimp STAT by 59.1% compared with the control, respectively (**Figure 7E**). Besides, overexpression of LvPTPN6 significantly promoted the dimerization of STAT, the essential procedure of STAT activation (**Figure 7F**). These data indicated that LvPTPN6 could facilitate the activation of the JAK/STAT signaling pathway.

### Involvement of LvPTPN6 in Antiviral Immunity

**Figure 8:**
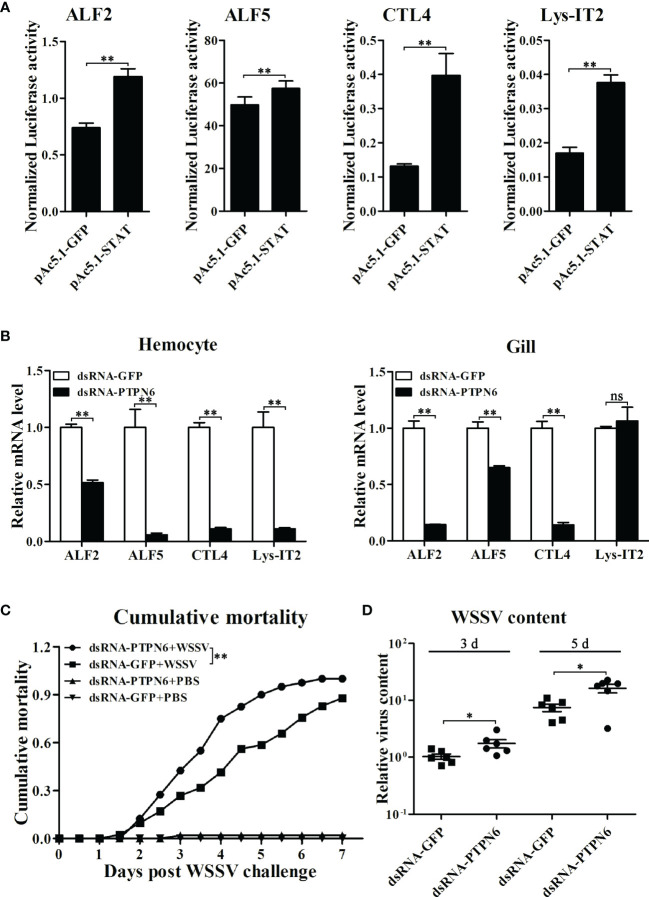
Role of LvPTPN6 in shrimp antiviral immunity. **(A)** Regulatory effects of shrimp STAT on the promoter of immune effector genes. Each bar represents the mean ± SD (*n* = 8), **p < 0.01 by two-tailed unpaired Student’s *t*-test. **(B)** qRT-PCR analysis of the mRNA level of STAT-regulated immune effector genes in hemocyte and gill at 48 h after dsRNA injection. Values in the dsRNA-GFP control group were set as the baseline (1.0). Each bar represents the mean ± SD (*n* = 4), **p < 0.01 and *ns*: *P* > 0.05 by two-tailed unpaired Student’s *t*-test. **(C)** Cumulative mortality of dsRNA-injected shrimps (*n* = 40) after WSSV infection. Data were recorded every 6 h and statistically analyzed by Kaplan–Meier log-rank χ^2^ test, **p < 0.01. **(D)** The relative viral load in muscle was analyzed by detecting the DNA of the WSSV *ie1* gene in six randomly selected shrimps using qRT-PCR with three repetitions, and the shrimp EF-1α gene was used as the internal control. The level in dsRNA-GFP group at 3 days post WSSV infection was set as baseline (1.0). *p < 0.05 by two-tailed unpaired Student’s *t*-test.

Before investigating the regulatory effects on the expression of immune effector genes mediated by LvPTPN6, the transcriptional activation of STAT on the promoters of *L. vannamei* immune effector genes was investigated by dual-luciferase assays. Results revealed that STAT could significantly activate the transcription of ALF2, ALF5, CTL4, and Lys-IT2 (**Figure 8A**). Correspondingly, the expressions of ALF2, ALF5, CTL4, and Lys-IT2 were generally suppressed in hemocyte and gill after knockdown of LvPTPN6 *in vivo* (**Figure 8B**). The antiviral role of LvPTPN6 was further investigated in shrimp. After knockdown of LvPTPN6, the cumulative mortality of shrimps after 3 days of WSSV infection was significantly increased compared with the control (**Figure 8C**), and the difference between the two treatments reached a peak of 33.5% at 4 days post WSSV infection. Consistently, the virus load of WSSV in muscles was significantly increased in the LvPTPN6-silenced group at 3 and 5 days post WSSV infection (**Figure 8D**). These data indicate that LvPTPN6 could facilitate antiviral immunity through the JAK/STAT singling pathway in shrimp.

## Discussion

Tyrosyl phosphorylation plays a critical role in multiple signaling pathways regulating innate and acquired immunity, which is mainly regulated by the dynamic equilibrium between protein-tyrosine kinases (PTKs) and protein-tyrosine phosphatases (PTPs) (3). Non-receptor PTPs are a classical type of PTPs, which consist of several subtypes distinguished with different domain features (11). SH2-domain-containing PTPs (SHPs) are characterized by composition of an N-terminal SH-2 domain, a C-terminal SH-2 domain, a classical PTP domain, and a C-terminal tail that contains two tyrosyl phosphorylation sites (36). In mammals, there are two kinds of SHPs, SHP1 (PTPN6) and SHP2 (PTPN11), which possess similar overall structures and regulatory mechanisms but differ in detailed structure, expression pattern, and, most importantly, physiological functions (3). In this study, an SH2 domain-containing PTP (LvPTPN6) sharing a higher homology with PTPN6 from other animals was identified in *L. vannamei*, which contains the classical domain features of SHPs.

Different from the distribution characters of human PTPN6 which is most abundantly expressed in hematopoietic cells and low in some epithelial, endothelial, and central nervous system cells (37), LvPTPN6 exits in all detected tissues. Besides, human PTPN6 is most abundantly expressed in the nucleus of epithelial cells and the cytoplasm of hematopoietic cells (38), while the subcellular localization analysis in S2 cells showed that LvPTPN6 was mainly located in the cytoplasm. These features corroborate the assertion that LvPTPN6 may have specific functions that differ from those of mammalian PTPN6s.

In addition to the posttranscriptional approaches, such as truncation, phospholipid binding, or tyrosine phosphorylation of the C-terminal tail, the activity of mammalian PTPN6 can also be regulated from transcriptional levels (39, 40). In the current study, LvPTPN6 was transcriptionally regulated after the challenge of stimulants, which share different expression patterns in hemocyte and gill, implying its complicated regulatory role in the shrimp immune responses.

In the canonical IFN systems, IRF activates the JAK/STAT signaling pathway to establish the cellular antiviral state through regulating the secretion of IFNs recognized by INF receptors and initiating the phosphorylation cascade of JAK and STAT (12, 28, 41). The IRF/Vago/JAK-STAT axis in shrimp is known to be similar to the interferon system of mammals and also plays an essential role in antiviral response (26, 42). Except the extracellular signal axis mediated by IFNs, very few studies revealed the intracellular activation of the JAK/STAT pathway regulated by IRF. For instance, in children with autosomal recessive homozygous IRF9 deficiency, IFN-stimulated response element (ISRE)-regulated transcription was attenuated for the disruption of the STAT1/STAT2/IRF9 heterotrimer; this finding presented an instance of IRF that could directly facilitate the transcriptional activation function of STAT (43). In this study, we found that IRF could activate the expression of LvPTPN6, further facilitating the nuclear localization of STAT and promoting the antiviral immunity of shrimp. Our results revealed that in addition to the canonical IRF/Vago/STAT axis, LvPTPN6 mediated an alternative regulatory approach from IRF to STAT. This novel regulatory strategy between IRF and STAT may provide a basis for further research on the IFN systems.

The JAK/STAT pathway is one of the important immune signaling pathways and exists wildly from vertebrates to invertebrates, which is regulated by different posttranscriptional mechanisms including acetylation, demethylation, and serine or tyrosine de-/phosphorylation (21). One of the major mechanisms that attenuate JAK/STAT pathway activation is dephosphorylation of the tyrosine residues by PTPs (22). In mammals, most of the PTP members, such as PTP receptor-type D (PTPRD), PTP receptor-type T (PTPRT), PTP receptor-type K (PTPRK), PTPN2, PTPN6, PTPN9 and PTPN11, had been proved to suppress the signal transduction of the JAK/STAT pathway by dephosphorylating on JAKs or STATs (44–50). Accumulating evidence has established the important role of PTPN6 in the regulation of the JAK/STAT signaling pathway in different organisms (51). The absence of SPH1 (PTPN6) activates JAK/STAT signaling pathways *via* the enhanced phosphorylation of JAK1, JAK2, JAK3, STAT3, STAT5, or STAT 6 (52), while its induction by chemical compounds could reduce the phosphorylation of STAT3, STAT5, or STAT 6 to block its signal transduction (22). Interestingly, our present finding demonstrated that LvPTPN6 could promote the unclear localization of STAT, activate the expression of STAT-regulated genes, and ultimately initiate the antiviral status of shrimp. Although most of the PTPs exhibit negative regulatory roles through their dephosphorylation activity in the JAK/STAT signaling pathway, some exceptions can promote it in specific circumstances. For example, the EGF- and IFN-γ-induced STAT activation was suppressed by expressing a catalytically inactive form of SHP1 (PTPN6) in HeLa cells, while overexpression of the native SHP1 had no effect on EGF-induced STAT activation but showed a positive effect on IFN-γ-induced STAT activation. This suggests that SHP-1 can function as a positive regulator in the activation of STAT (23). Inhibition of SHP2 expression initially enhanced and later inhibited STAT5 phosphorylation and reduced the expression of the antiapoptotic genes of MCL1 and BCLXL (53). In mouse mammary gland cells, SHP2 plays a positive role in the prolactin-induced JAK2 activation pathway. JAK2 tends to associate with suppressor of cytokine signaling 1 (SOCS1), which targets JAK2 through a ubiquitin-dependent degradation pathway and serves as a negative regulator for the JAK2/STAT5 pathway. The interaction between JAK2 and SOCS1 is mediated by phosphorylation of Tyr1007 in JAK2. *In vitro* studies demonstrated that SHP2 was able to dephosphorylate this Tyr site and prevent the formation of the JAK2-SOCS1 complex and subsequent degradation of JAK2. Upon being released from the inhibitory effects of SOCS1, JAK2 is recruited to the prolactin receptor (PrlR) and phosphorylates STAT5 (24). However, the precise mechanism of how these molecules achieve positive functions in different systems remains to be clarified. In this study, LvPTPN6 can both combine with JAK and STAT and elevate the dimerization of STAT, providing us with clues that LvPTPN6 may belong to catalytically inactive PTPs, which could enhance the stability of the JAK and STAT dimers by blocking their tyrosyl phosphorylation sites from other PTPs. To the best of our knowledge, it is the first report of a PTP positively regulating JAK/STAT pathway in invertebrates. These findings indicated that the structures and functions of PTPs vary from different organisms that required further excavation to reveal their non-negligible regulatory roles in invertebrates and vertebrates.

## Data Availability Statement

The original contributions presented in the study are included in the article/**Supplementary Material**. Further inquiries can be directed to the corresponding authors.

## Author Contributions

HZ and JH supervised the overall project and designed the experiments. HZ wrote the manuscript. ML and XX performed the experiments and analyzed the data with the help from XL, LY, WS, and SW. ZZ offered the assistance to revise the manuscript. All authors contributed to the article and approved the submitted version.

## Funding

This work was funded by the Natural Science Foundation of Guangdong Province, China, 2020A1515011152 and 2021A1515010798; National Natural Science Foundation of China under grant nos. 31972823, 32073004, and 31772881; National Key Research and Development Program of China, 2018YFD0900505; China Agriculture Research System CARS48; and Key-Area Research and Development Program of Guangdong Province, China, 2019B020217001.

## Conflict of Interest

The authors declare that the research was conducted in the absence of any commercial or financial relationships that could be construed as a potential conflict of interest.

## Publisher’s Note

All claims expressed in this article are solely those of the authors and do not necessarily represent those of their affiliated organizations, or those of the publisher, the editors and the reviewers. Any product that may be evaluated in this article, or claim that may be made by its manufacturer, is not guaranteed or endorsed by the publisher.

## Supplementary Material

Supplementary Figure 1 Sequence and domain analysis of LvPTPN6. **(A)** Nucleotide and deduced amino acid sequences of LvPTPN6. The nucleotide (lower case) and deduced amino acid (upper case) sequences were shown and numbered on the left. The putative SH2 domains were framed and the PTPc domain was shadowed. **(B)** Structural domains of LvPTPN6 predicted by SMART (http://smart.embl-heidelberg.de/).

Supplementary Figure 2 Sequence and the predicted IRF binding motif of LvPTPN6 promoter. **(A)** Nucleotide sequence of LvPTPN6 promoter. The predicted IRF binding motif was framed, and the cleave site of the cleaved promoters mentioned in FIGURE 5 B were marked by black bars. **(B)** Results of *Homo sapiens* IRF2 binding motif in LvPTPN6 promoter predicted by JASPAR (http://jaspar2016.genereg.net/cgi-bin/jaspar_db.pl).

Supplementary Figure 3 Microscopic vision fields for the immunofluorescent intensities analysis of **Figure 7C**.
