# Supplementary figures and images for: The Non-Receptor Protein Tyrosine Phosphatase PTPN6 Mediates a Positive Regulatory Approach From the Interferon Regulatory Factor to the JAK/STAT Pathway in Litopenaeus vannamei
Source: Front Immunol. 2022 Jun 29;13:913955. doi: 10.3389/fimmu.2022.913955 (PMC9276969; doi:10.3389/fimmu.2022.913955)

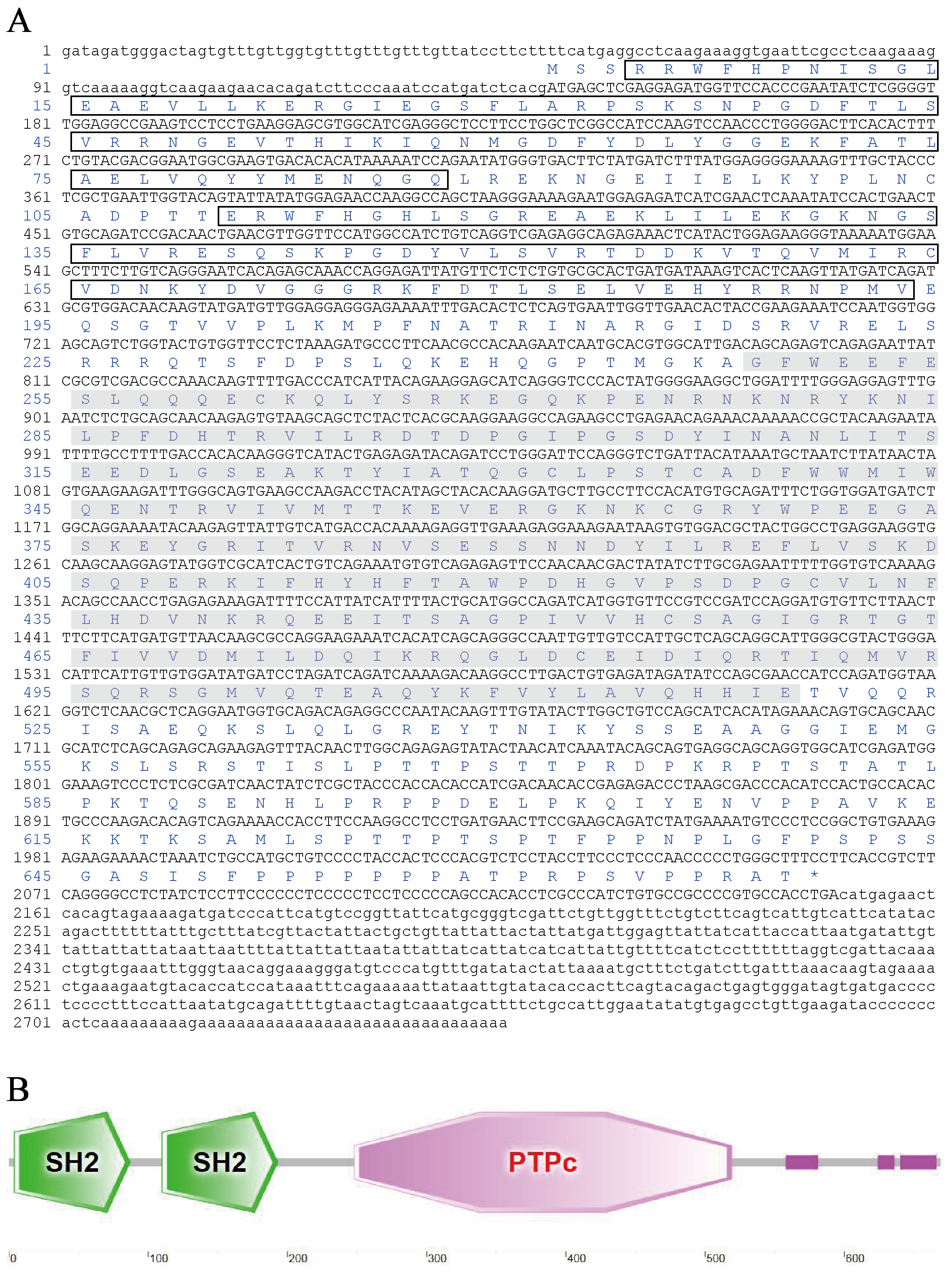

Supplement: Supplementary Figure 1 — Sequence and domain analysis of LvPTPN6. (A) Nucleotide and deduced amino acid sequences of LvPTPN6. The nucleotide (lower case) and deduced amino acid (upper case) sequences were shown and numbered on the left. The putative SH2 domains were framed and the PTPc domain was shadowed. (B) Structural domains of LvPTPN6 predicted by SMART (http://smart.embl-heidelberg.de/). [file Image_1.tif]

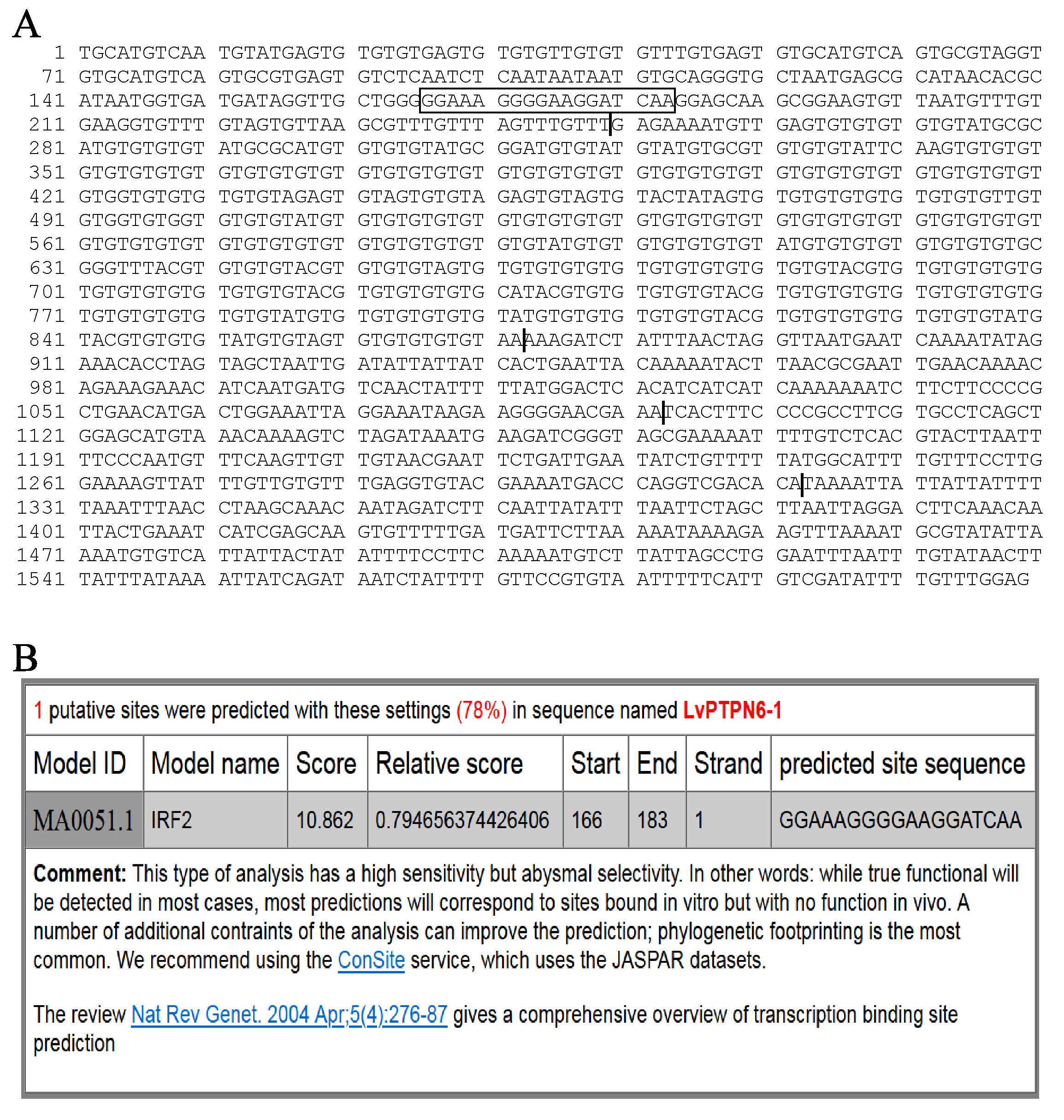

Supplement: Supplementary Figure 2 — Sequence and the predicted IRF binding motif of LvPTPN6 promoter. (A) Nucleotide sequence of LvPTPN6 promoter. The predicted IRF binding motif was framed, and the cleave site of the cleaved promoters mentioned in FIGURE 5 B were marked by black bars. (B) Results of Homo sapiens IRF2 binding motif in LvPTPN6 promoter predicted by JASPAR (http://jaspar2016.genereg.net/cgi-bin/jaspar_db.pl). [file Image_2.tif]

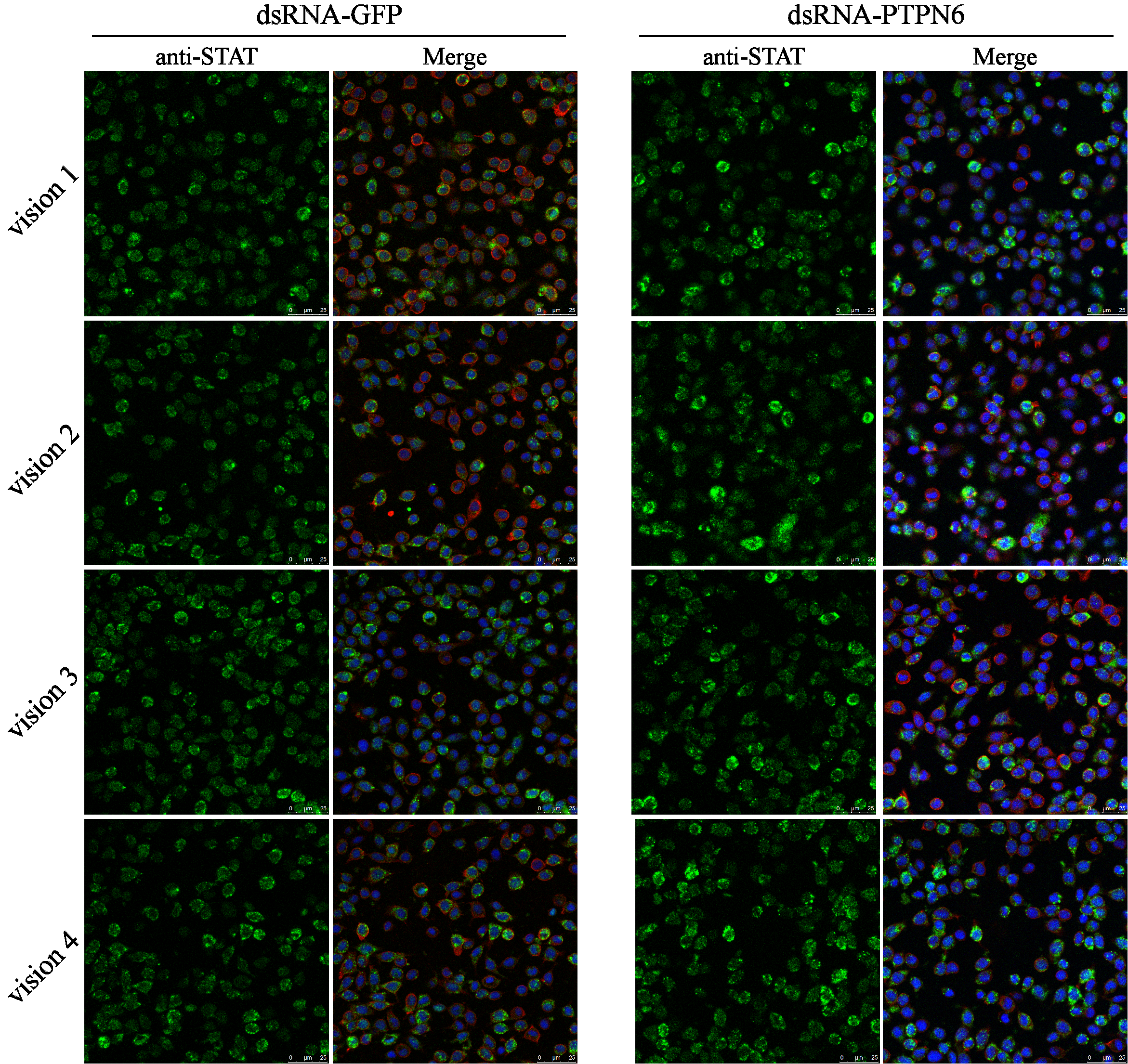

Supplement: Supplementary Figure 3 — Microscopic vision fields for the immunofluorescent intensities analysis of Figure 7C . [file Image_3.tif]
